# Regional land use and land-use intensity effects on vertebrate biodiversity across Europe identified using species distribution models

**DOI:** 10.1007/s10980-026-02379-y

**Published:** 2026-05-18

**Authors:** Sophie Jane Tudge, Adrienne Etard, Zoe M Harris, Adriana De Palma, Martin Jung

**Affiliations:** 1 Biodiversity, Ecology, and Conservation Research Group, International Institute for Applied Systems Analysis, Schlossplatz 1, AT 2361 Laxenburg, Austria; 2 Faculty of Engineering and Physical Sciences, Centre for Environment and Sustainability, University of Surrey, Guildford, GU2 7XH UK; 3 Institute for Sustainability, University of Surrey, Guildford, GU2 7XH UK; 4 Biodiversity Futures Lab, Research Department, Natural History Museum, London, SW7 5HD UK

**Keywords:** Europe, Habitat suitability, Land use, Land-use intensity, Species distribution model, Vertebrate

## Abstract

**Context:**

Land-use change and intensification are major drivers of biodiversity loss. However, estimates of the effects of land use and land-use intensity on biodiversity on broad spatial scales rarely account for species-specific responses across large areas, instead averaging effects on local assemblages. Current understanding of biodiversity effects on broad spatial scales is therefore limited.

**Objective:**

To investigate the broad-scale effects of land use and land-use intensity on habitat suitability for cropland and forest vertebrates throughout their contemporary ranges in Europe. To summarise regional-scale effects on vertebrate biodiversity patterns in Europe by synthesising the effects on range-wide habitat suitability for individual species.

**Methods:**

Cropland and forest vertebrate occurrence records and geographic range estimates were combined with a European land-systems map. Species distribution models (SDMs) were used to estimate the effects of land use and land-use intensity on habitat suitability on a landscape scale throughout species’ ranges. Linear coefficients were specified to keep models interpretable, avoid overfitting and facilitate the use of priors based on expert knowledge. Regional-scale effects were explored for species groups based on habitat preferences, taxonomic groups and range sizes.

**Results:**

Land use and land-use intensity significantly affected cropland and forest vertebrate habitat suitability on a landscape scale across Europe. The effects of land use and land-use intensity on habitat suitability varied among species groups. Land‑use intensification and urbanisation disproportionately favoured more widespread species.

**Conclusions:**

Landscape-scale land use and land-use intensity help shape regional biodiversity patterns across Europe via their influence on habitat suitability throughout species’ contemporary ranges.

**Supplementary Information:**

The online version contains supplementary material available at 10.1007/s10980-026-02379-y.

## Introduction

Global biodiversity is in decline, with approximately one quarter of animal and plant species threatened with extinction (IPBES 2019). Land-use change – or the change in use of land by humans – is among the leading causes of terrestrial biodiversity loss, predominantly driven by agricultural expansion, commercial forestry and urbanisation (IPBES 2019). The negative effects of land-use change on biodiversity are usually associated with the loss and fragmentation of natural vegetation (Haddad et al. 2015; Powers and Jetz 2019). Land-use intensification, however, refers to changes in the management of a particular land use that increase land productivity, and usually decrease biodiversity (e.g. Newbold et al. 2015, Beckmann et al. 2019, Semenchuk et al. 2022, Van 'T Veen et al. 2025). While there are different ways to define land-use intensity, it is generally measured in relation to inputs (e.g. fertilisers, pesticides or irrigation), outputs (e.g. yield) or land-system properties (e.g. disturbance regimes) (Erb et al. 2013; Kehoe et al. 2015; Dullinger et al. 2021). Land-use intensification modifies the suitability of different land uses for biodiversity (Semenchuk et al. 2022), but land-use intensity is often overlooked in models of the effects of land use on biodiversity (Dullinger et al. 2021). In the future, both intensification and de-intensification (e.g. agricultural land abandonment or restoration) are likely to occur (Stehfest et al. 2019). Therefore, the exclusion of land-use intensity from models predicting the effects of land-use change on biodiversity could result in under- or over-estimation of biodiversity trends (Kehoe et al. 2015; Dullinger et al. 2021).

Current knowledge of the effects of land use and land-use intensity on biodiversity typically comes from spatial comparisons of local assemblages (e.g. Semenchuk et al. 2022; Zhao et al. 2024). Such comparisons have identified negative effects of land-use change and intensification on, for example, bird taxonomic diversity (Hevia et al. 2016) and herpetofauna abundance (Falaschi et al. 2019) in Europe. However, land-use changes can affect biodiversity to different extents depending on spatial scale (e.g. Flohre et al. 2011), such that local-scale diversity increases can co-occur with broader-scale diversity decreases (Gaüzère et al. 2025). Furthermore, averaging the results of local-scale biodiversity assessments over larger areas can lead to underestimations of biodiversity loss on broader scales (Socolar et al. 2025). For example, there is evidence that land-use changes may cause more severe biotic homogenisation at regional rather than local spatial scales; yet, few studies directly assess regional-scale effects (Socolar et al. 2025). Indeed, relatively little is understood about the spatial scale at which species respond to land-use changes, despite its importance for predicting species’ vulnerabilities to future changes (Duncan et al. 2020).

Other sources of variation in the response of biodiversity to land-use change and intensification also exist. For example, different taxonomic and functional groups of species can show different responses (e.g. Wood et al. 2017; Beckmann et al. 2019; Graham et al. 2019). Importantly, there are indications that intensification may be linked to a disproportionate loss in habitat specialists (e.g. Šálek et al. 2018; Rédei et al. 2020; Kuipers et al. 2023; Etard and Newbold 2024) and species with smaller range sizes (Newbold et al. 2018; Etard and Newbold 2024). An associated shift in community composition towards more geographically widespread species can result in increased similarity between communities across space, with negative consequences for the conservation of rare species (Olden & Rooney 2006; Ripple et al. 2017). Previous reviews also suggest that vertebrates may be more resilient to intensification in wood- rather than crop-production systems (Beckmann et al. 2019; Cordeiro Pereira et al. 2024; Jones et al. 2024). Lastly, vertebrates are also affected by the availability of suitable habitats on a landscape-scale; thus, landscape structure can influence the effects of land-use change and intensification on biodiversity (Gonthier et al. 2014; Le Provost et al. 2021). Therefore, consideration of variation in the effects of land use and land-use intensity on different species groups, especially on broad spatial scales, is important for conservation.

Europe has a long history of intensive land use and related losses in biodiversity (Pereira et al. 2024). For example, the EU 27 + region contains relatively little land that has not been modified by humans (Sandström et al. 2023). Since 1900, urbanisation, afforestation and cropland-grassland transitions have been among the most important land-use changes to occur (Fuchs et al. 2015). Additionally, most land uses (including cropland, forest, grassland and urban) are managed intensively. For example, the EU 27 + region contains less than 1% by area of primary forest and low-intensity arable cropland combined, but approximately 10% by area of intensively-managed forest and 13% high-intensity arable cropland (Sandström et al. 2023). Therefore, due to the region’s land-use history, extant European species may be relatively tolerant of land-system pressures (Marjakangas et al. 2024). For example, low-intensity management can sometimes enhance biodiversity, such as with low levels of fertilisation in nutrient-poor grasslands (Dengler et al. 2020).

However, cropland species in particular have been declining in Europe due to agricultural intensification (Reif and Vermouzek 2019; Rigal et al. 2023). Forest species are also subject to increasing management intensity, with potential implications for the persistence of affected populations of such species (Sabatini et al. 2020; Jung et al. 2022). Yet, target 10 of the Kunming-Montreal Global Biodiversity Framework, to which many European countries are committed, focuses specifically on increasing biodiversity and sustainability in agriculture and forestry (CBD 2022). Furthermore, the EU Nature Restoration Regulation, which aims to reverse biodiversity declines across the EU, includes specific targets to increase farmland and forest bird populations, chosen as indicators of the biodiversity status of agricultural and forest habitats (Regulation (EU) 2024/1991). These targets are in place against a backdrop of projected changes in land use and land-use intensity over the coming decades (Alexander et al. 2017; Visconti et al. 2024), which could affect species’ habitat availability (e.g. Finch et al. 2023). Therefore, a more detailed assessment of the effects of changes in land use and land-use intensity on cropland and forest vertebrates in Europe, on a broad spatial scale, could help with strategic land-use planning that accounts for impacts on these species.

Species distribution models (SDMs) are widely used to assess how environmental – mainly climatic – variables influence the likelihood of occurrence of species, and thus to estimate species’ distributions in space and time. Yet, research into how non-climatic human predictors – including land-use variables – affect species distributions is rare (Titeux et al. 2016; Milanesi et al. 2020; Frans and Liu 2024). Additionally, SDM-based approaches focused on understanding the effects of landscape-scale changes on multiple taxa, over large spatial extents, are scarce (Pearman-Gillman et al. 2020). Nevertheless, some recent studies have demonstrated the applicability of land-use predictors to SDMs (Marshall et al. 2021; Gábor et al. 2024), and several studies have used land-use predictors to help understand changes in the availability and distribution of suitable habitat for vertebrate species (e.g. Cardador and Blackburn 2019; Pearman-Gillman et al. 2020; Devenish et al. 2021; Davoli et al. 2024). Consideration of land-use intensity, however, is even rarer than land use, likely because of the challenge of obtaining meaningful coefficients (Duncan et al. 2020; Chapman et al. 2025).

In this study, we used a novel methodological approach – based on SDMs – to estimate the broad-scale effects of landscape-scale land use and land-use intensity on habitat suitability for cropland and forest vertebrates across Europe. Our data comprised biodiversity occurrence records from the literature, a recent fine-resolution land-system map (Sandström et al. 2023) and expert-derived species-ecosystem associations (Roscher et al. 2015). We used the ibis.iSDM modelling framework (Jung 2023) and the area of habitat – or extent of suitable habitat – concept (Brooks et al. 2019) to model habitat suitability throughout species’ contemporary ranges. Large-scale SDM-based studies rarely use existing knowledge about species habitat preferences to constrain model predictions, despite the potential for ecological knowledge to help improve model realism (Gaget et al. 2025). For example, while ecological knowledge is primarily used for variable selection, using it to directly influence predictor coefficients can help to strengthen patterns based on species occurrence data alone (Murray et al. 2009) and make more ecologically plausible models (Hofner et al. 2011). Expert ecological knowledge, in particular, can be useful for making informed priors in SDMs that guide and constrain predictor coefficients (Murray et al. 2009), but it has thus far been underused for inferring the effects of land-use and land-use intensity.

Here, we used the best available expert knowledge of species-ecosystem associations that we could find, provided by the European Environment Agency (Roscher et al. 2015), to make informed priors for each species. Priors were based on the known suitability of different habitats and were used to derive more ecologically realistic response functions. We were interested in assessing the responses of species to land use and land-use intensity within their contemporary ranges, and not in predicting species’ distributions. However, a model validation approach that is based on response functions rather than spatial predictions is currently lacking. Such an approach would require not only unbiased testing data, but also a way to include expert knowledge (e.g. priors) in the validation. Thus – in contrast to previous studies – we focused on the robustness of estimated statistical relationships to assess model performance, rather than using traditional spatial model validation metrics. We explored variability in the partial effects of land use and land-use intensity on habitat suitability according to species’ known habitat preferences, taxonomic groups and geographic range sizes, given previous indications that these variables could be important (Graham et al. 2019; Etard and Newbold 2024). We hypothesised that 1) landscape-scale land-use and land-use intensity predictors would significantly affect vertebrate habitat suitability throughout species’ contemporary ranges, and 2) the relative suitability of different land uses – and the effects of intensification – would differ according to species’ habitat preferences, taxonomic groups and range sizes.

## Methods

### Biodiversity data

We collated occurrence records and geographic ranges for terrestrial vertebrates from a range of data sources, using the following criteria:

1. The species occurred within the European areas mapped by Sandström et al. (2023),
2. The species had at least one of “cropland” and “woodland and forest” as a preferred ecosystem, according to expert knowledge gathered for species of the Habitats and Birds Directives and held within a database detailing species’ associations with ecosystem types (Roscher et al. 2015),
3. The species had at least 100 occurrence records since 2000 in the Global Biodiversity Information Facility (GBIF 2024) and/or eBird (Sullivan et al. 2009), chosen as a compromise between avoiding small sample sizes and maximising the number of species included (Santini et al. 2021), and
4. The species’ range was available from the IUCN Red List (IUCN 2024), BirdLife International Important Bird Areas (BirdLife International 2006) or reporting for the EU Habitats (Council Directive 92/43/EEC) and/or Birds Directive (Directive 2009/147/EC). Alternatively, the species had been recorded in at least one Natura 2000 site checklist (Council Directive 92/43/EEC).

Occurrence records from GBIF were filtered to remove those with missing or uncertain metadata (including occurrences where coordinate uncertainty was > 5 km, the coordinates intersected water or where there were other reported issues) using the “CoordinateCleaner” R package (Zizka et al. 2019). Occurrence records were pooled into one dataset per species. eBird records were considered presence-only observations for consistency with the GBIF records. As the land-use and land-use intensity predictors were only available for the European areas mapped by Sandström et al. (2023), we only considered contemporary range distributions of species within these areas. The “st_area()” function from the “sf” R package was used to calculate the geographic range size (m^2^) of each species (Pebesma 2018). If a species’ range consisted of multiple non-overlapping polygons, from one or more data sources, total range size was calculated as the sum of the areas of all individual polygons. We then categorised species ranges into small-sized (≤ 33rd percentile, or ≤ 5.76 × 10^11^ m^2^), medium-sized (33rd to 66th percentile, or 5.76 × 10^11^ to 1.71 × 10^12^ m^2^) or large-sized (≥ 66th percentile, or ≥ 1.71 × 10^12^ m^2^), based on the distribution of range sizes of the species included. On a global – rather than a European – scale, the range-size groups could therefore be different. Additionally, other methods of delimiting species’ ranges, such as by joining non-overlapping polygons to form contiguous areas, could also affect calculations of range size and range-size groups. Species were grouped taxonomically into mammals, birds and herpetofauna, and each species’ European Regional IUCN Red List Category was taken from IUCN (2024). All further analyses were conducted in R version 4.3.1 (R Core Team 2023).

### Land-use and land-use-intensity data

We used the 1 km^2^ resolution land-system map (v3) from Sandström et al. (2023), which distinguished land systems into 20 land-use and land-use-intensity classes. Classes that had low spatial coverage were combined to increase overlap with species ranges and enable more robust estimation of their effects. We thereby obtained 11 classes and re-coded each pixel to zero (not present) or one (present) at 1 km^2^ resolution. Then each binary layer was aggregated to 5 km^2^ resolution using the arithmetic mean, resulting in 11 continuous rasters representing the fractional share (proportional cover) of each land-use or land-use-intensity class within each 5 km^2^ pixel (Table 1). This approach captured broader landscape-scale effects – which have been shown to be important for modelling vertebrate species distributions (e.g. Devenish et al. 2021) – and reduced computation time. Additionally, proportional land-use cover variables have been recommended for SDMs at > 1 km^2^ resolution (Gábor et al. 2024). We categorised cropland, forests, grassland and urban areas each into low and high land-use intensity. Ideally, these land uses would each have retained three land-use-intensity classes. However, imbalance in the spatial coverage of each intensity necessitated combining at least two intensities together for each land use. Combining the low- and medium-intensity categories and retaining the high-intensity categories resulted in the best spatial balance between the resultant categories, allowed for more focus on the effects of high-intensity land use, and eased interpretation and comparison of the results. See Online Resource 1 for further details on land-use intensity differences.

**Table 1:** Re-classification of land-use and land-use-intensity classes from Sandström et al. (2023) into land-use and land-use-intensity classes used in this study, which were inferred to be suitable habitats based on the corresponding ecosystem association data from Roscher et al. (2015). Mosaic habitats did not have a corresponding ecosystem in Roscher et al. (2015), so no inferences were made about the suitability of mosaic habitats for species

Land-use or land-use-intensity class(es) from Sandström et al. (2023)	Land-use or land-use-intensity class used in this study	Suitable ecosystem(s) based on Roscher et al. (2015)
Water and glaciers; wetland	Water and wetland	Wetlands; marine inlets and transitional waters; rivers and lakes
Bare, rock and shrub	Bare, rock and shrub	Sparsely vegetated land
Forest, shrub and cropland mosaic; forest, shrub and grassland mosaic	Mosaic	N/A
Low-density rural settlement; medium-density peri-urban settlement	Low-intensity urban	Urban
High-density urban settlement	High-intensity urban	Urban
Unmanaged forest nature reserve; close-to-nature-forestry; combined-objective forestry	Low-intensity forestry	Woodland and forest
Intensive-even-aged-forestry; short-rotation forestry	High-intensity forestry	Woodland and forest
Low-intensity grassland; medium-intensity grassland	Low-intensity grassland	Grassland
High-intensity grassland	High-intensity grassland	Grassland
Low-intensity arable cropland; medium-intensity arable cropland; permanent cropland	Low-intensity cropland	Cropland
High-intensity arable cropland	High-intensity cropland	Cropland

### Species-ecosystem associations

We used expert ecological knowledge of species-ecosystem associations from Roscher et al. (2015) to infer species’ habitat preferences, and to determine the suitability of land uses for each species. The species-ecosystem associations were later used in the modelling process to specify informed priors that constrained the effect of a suitable land use for a species to be positive (i.e. the model coefficient for that land use could not be < 0) (Hofner et al. 2011, Fletcher Jr. et al., 2019, Jung 2023), and to spatially define likely unsuitable habitat for pseudoabsence sampling. A species could have more than one suitable or unsuitable land use (we determined a suitable land use as one where the corresponding ecosystem was listed as either preferred, occasional or suitable). Table 1 shows the linkages between ecosystem associations and suitable land uses. A preferred ecosystem was the most important ecosystem for a species according to Roscher et al. (2015), which was usually used for the life cycle of the species or was linked to the largest population of the species. Thus, we summarised our model results for cropland-preferring (hereafter called cropland) and forest-preferring (forest) species separately.

### Species distribution modelling

We tested for multicollinearity in our land-use and land-use-intensity variables using the “usdm” R package (Naimi 2023). All variance inflation factors were < 1.3; thus, collinearity was not deemed problematic (Dormann et al. 2013) and all variables were used as predictors in our models. We constrained model parameterisation to within each species’ contemporary range, assuming that species ranges broadly reflected species climatic niches, as we were specifically interested in the current relative suitability of different land uses and land-use intensities within each species’ range. We did not include other predictors in our models to increase the interpretability of our results with respect to land use and land-use intensity (Merow et al. 2014). Although including other predictors – such as bioclimatic variables – could result in better spatial predictions of species distributions (Marshall et al. 2021; Tsiftsis et al. 2024), our focus was on identifying the statistical relationships between land use, land-use intensity and habitat suitability within species contemporary ranges (Araújo et al. 2019).

To improve SDM accuracy, we generated pseudoabsence points within each species’ range, the number of points being equal number to the number of presence points gathered for each species (Barbet-Massin et al. 2012). We geographically restricted pseudoabsence sampling to land uses in Sandström et al. (2023) that were deemed unsuitable for each species – based on expert knowledge – following the species-ecosystem association methodology described above. If no land uses were unsuitable for a species, we randomly generated pseudoabsence points throughout the species’ range. As we were only interested in species presence or pseudoabsence, we thinned observations for each species to ≤ 1 per pixel (i.e. present or not). Additionally, we looked for potential sampling biases in the biodiversity data by assessing the proportion of occurrence points that fell within each land-use and land-use-intensity class.

No single SDM method is best suited to all species and situations (Valavi et al. 2022). Here, we wanted to explicitly include informed priors on predictor coefficients in our models, which is not possible using, for example, the common MaxEnt method (Elith et al. 2011). Coefficient priors can counterbalance the effects of biased SDM predictions, which can arise due to environmental and spatial bias of occurrence points (Santini et al. 2021), by constraining coefficients in line with prior knowledge of species ecology. Therefore, coefficient priors can help to derive more ecologically realistic response functions. Thus, we used the “ibis.iSDM” R package version 0.1.5 to fit our SDMs, which provides a flexible framework with convenient functions for integrating data in SDMs, including for specifying priors (Jung 2023; Jung and Hesselbarth 2023). Within the ibis.iSDM package we used the “glmnet” engine (Friedman et al. 2010), which allows for the addition of penalisation priors on coefficients.

We focused on linear relationships to avoid overfitting and to strike a balance between data availability, model complexity and interpretability (García-Callejas and Araújo 2016; Duguma et al. 2023). Additionally, rather than using variable selection methods to select the best model for each species, we used regularisation, which is a more risk-averse method (Reineking and Schröder 2006) that can result in better-performing models (Valavi et al. 2022). Regularisation aims to reduce the complexity of models and avoid overfitting, prioritising generalisability and reliability (Reineking and Schröder 2006). We used ridge regularisation, which penalises and shrinks variables towards zero. However, we used the informed priors to specify that coefficients for suitable land uses could not be regularised. Reineking and Schröder (2006) found that regression models with ridge regularisation could be expected to perform well under a range of species-habitat relationships; thus, they were well suited to our multi-taxa, regional-scale assessment.

Therefore, for each species, we fitted regularised linear regression models with ridge regularisation using the glmnet engine within the ibis.iSDM R package. Informed priors for suitable land uses were added to each species’ model, specifying that the coefficients for suitable land uses could not be negative and could not be regularised out (Jung and Hesselbarth 2023), with the aim of minimising non-sensical response functions and aiding model inference. From each fitted SDM, we extracted model coefficients for each predictor. Further, across all species, we fitted additional linear models to test whether our predictors significantly affected the occurrence rate of species, i.e. inferred habitat suitability, and to test for significant differences in predictor coefficients due to land-use intensity and per species group (taxonomic group and range-size group).

Effective evaluation of SDM performance remains challenging, and development of reliable methods that prioritise the objectives and ecological relevance of results are necessary (Fourcade et al. 2018). Limitations of traditional model validation metrics, such as the commonly used area under the receiver operating characteristic curve (AUC), mean that accurate models can have low spatial predictive performance, especially for widespread species (Lobo et al. 2008; Tessarolo et al. 2014). Conversely, low model spatial predictive performance according to AUC does not necessarily invalidate estimates of the relative effects of model predictors, or their ecological importance, and does not imply an inaccurate assessment of habitat suitability (Lobo et al. 2008) (see Online Resource 1 for further discussion).

Here, we focused on the robustness of estimated predictor effects, rather than on model spatial predictive accuracy or generalisability outside the modelled system (Araújo et al. 2019), in line with the objectives of our study. To estimate the robustness of estimated effects, we assessed model coefficient stability (i.e. the reliability of the estimated effects of each land-use and land-use-intensity class) using spatial block cross-validation with five spatially separated blocks and five folds of the data, made with the “blockCV” R package (Valavi et al. 2019). For each species, we compared the magnitude and direction of the model coefficients for each predictor in each cross-validation model (using four folds of the data, withholding one fold for an evaluation of predictive performance at a later stage) with the full model. We then calculated, for each species, the coefficient of variation (CV) of the coefficient for each predictor, then the mean CV of all predictors. Mean CV was used as an indicator of the robustness of coefficient estimates, with values close to zero reflecting comparatively more stable coefficient estimates. Negative mean CV values indicated directional disagreement in model coefficients during cross-validation. We excluded from further analysis species for which the mean CV of coefficients was < 0 or exceeded the upper quartile of the distribution of values for all remaining species (1.06), due to having less confidence in the robustness of the estimates for those species. We used the withheld fold of data to generate statistics for the predictive performance of each cross-validation model using the “validate” function from the ibis.iSDM package, solely to aid interpretation in relation to the wider literature. For each species, we calculated the mean AUC of the cross-validation models (Pearce and Ferrier 2000), for which a threshold of ≥ 0.7 is typically used to indicate sufficient predictive accuracy (Van Proosdij et al. 2016).

## Results

We generated results for 229 species: 123 birds, 20 herpetofauna and 86 mammals (see Online Resource 2 for a species list and Online Resource 3 for species-land-use suitability). Seven species had negative values of mean CV and were excluded from further analysis. The average mean CV of model coefficients for the remaining 222 species was 0.61 (Online Resource 4). Of the 75% of remaining species (166 species) with the least variable coefficient estimates – whose results were thus considered the most robust – average mean CV of model coefficients was 0.40, including 112 birds, 12 herpetofauna and 42 mammals. Most of these species (73%) were forest-preferring (122 species), while 39% were cropland-preferring (65 species); both cropland and forest were preferred ecosystems for 21 species.

**Fig. 1:**
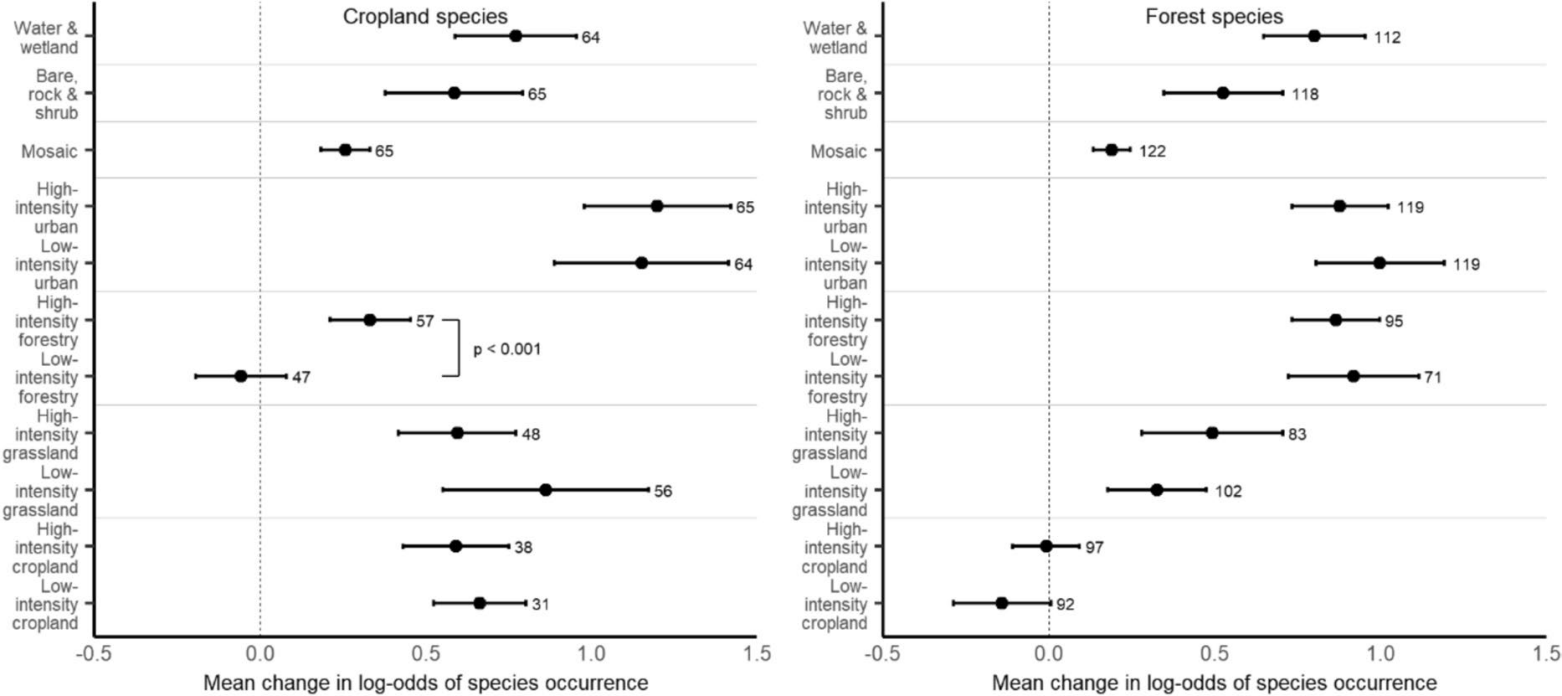
Mean change in log-odds of species occurrence for a one unit increase in land-use share of land-use and land-use-intensity SDM predictors for cropland and forest vertebrates across Europe. Predictor coefficients were interpreted as changes in log-odds of species occurrence. The magnitude of change in log-odds of species occurrence represents the importance of each predictor in relation to habitat suitability. Positive values means that the predictor had a positive effect on habitat suitability. Error bars represent 95% confidence intervals. Data point labels show the number of species included in each mean. Brackets represent a significant difference (*p* ≤ 0.05) between a land use of different use-intensity. Results are shown for the species with the most stable predictor coefficient estimates (0 ≤ species’ mean CV ≤ 1.06), as measured with cross-validation (*n* = 166)

**Fig. 2:**
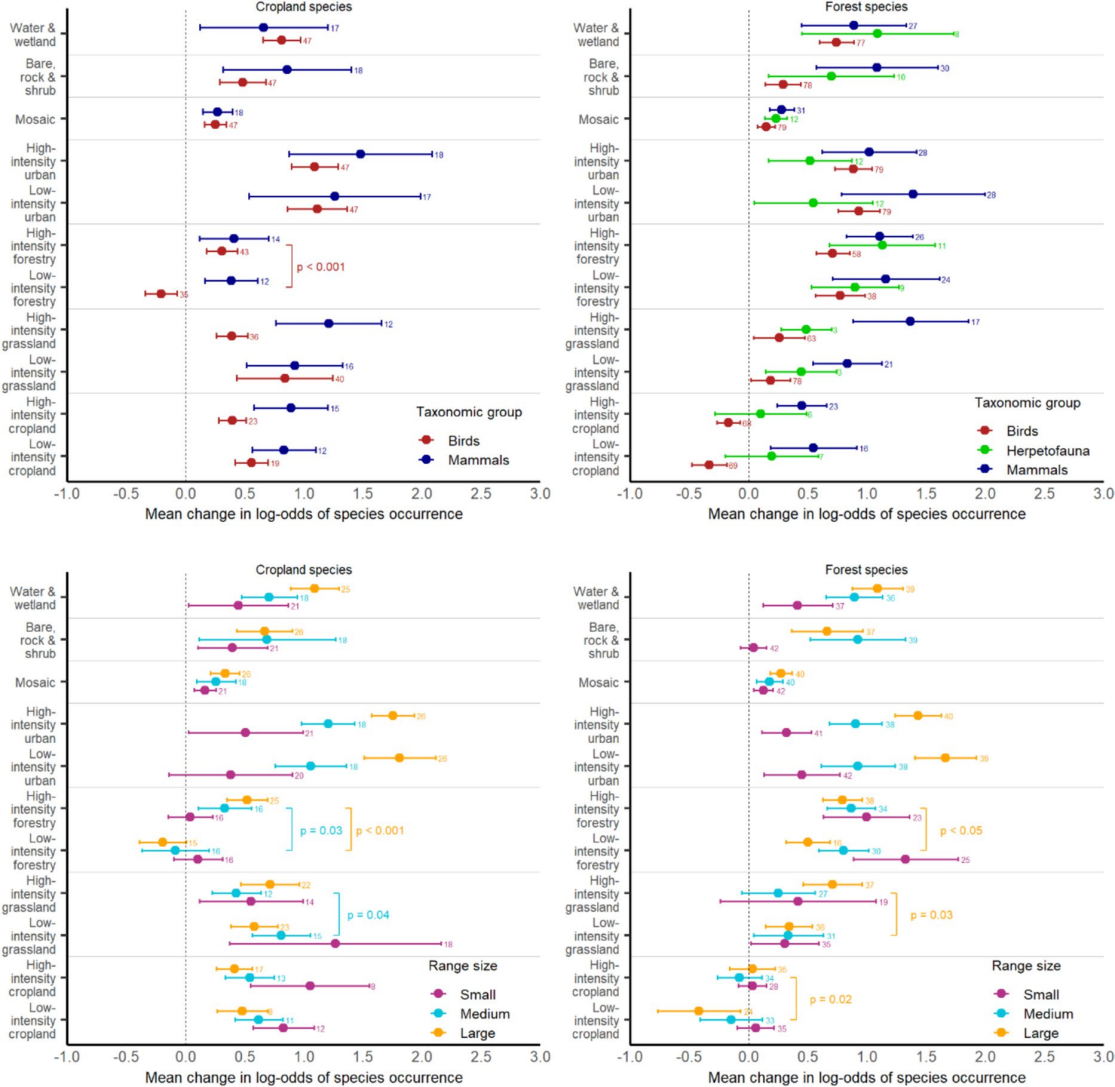
Mean change in log-odds of species occurrence for a one unit increase in land-use share of land-use and land-use-intensity SDM predictors for cropland and forest vertebrates across Europe, by taxonomic group and range size. Predictor coefficients were interpreted as changes in log-odds of species occurrence. The magnitude of change in log-odds of species occurrence represents the importance of each predictor in relation to habitat suitability. Positive values means that the predictor had a positive effect on habitat suitability. Error bars represent 95% confidence intervals. Data point labels show the number of species included in each mean. Brackets represent a significant difference (*p* ≤ 0.05) between a land use of different use-intensity. Results are shown for the species with the most stable predictor coefficient estimates (0 ≤ species’ mean CV ≤ 1.06), as measured with cross-validation (n = 166)

Overall, our predictors had significant effects on habitat suitability for cropland and forest species (Table 2). Urban was the most suitable land use for both cropland and forest species, on average (Fig. 1). However, our assessment of potential sampling bias did not find that most occurrence records originated from urban areas (Online Resource 5). Additionally, there were clear differences in the relative suitability of urban areas among range-size groups (Fig. 2). On average, for cropland species, cropland and grassland land uses of low land-use intensity were more suitable than the equivalent land uses of high intensity; however, the differences were non-significant. On the other hand, cropland species were significantly more likely to occur in high-intensity rather than low-intensity forestry, on average. For forest species on average, land-use intensity did not significantly affect habitat suitability for any land use.

**Table 2:** Results of six linear models that tested for the significance of the effects of land use and land-use intensity predictors on species’ habitat suitability, based on SDM predictor coefficients

Preferred ecosystem of species	Model structure	Model term	Degrees of freedom	F-value	Significance (*p*-value)
Cropland	Predictors	Predictors	11	24.84	< 0.001
	Predictors + taxonomic group + predictors: taxonomic group	Predictors	11	25.70	< 0.01
		Taxonomic group	1	10.47	< 0.001
		Predictors: taxonomic group	11	2.18	0.01
	Predictors + range size + predictors: range size	Predictors	11	28.77	< 0.001
		Range size	2	10.58	< 0.001
		Predictors: range size	22	4.82	< 0.001
Forest	Predictors	Predictors	11	42.00	< 0.001
	Predictors + taxonomic group + predictors: taxonomic group	Predictors	11	45.85	< 0.001
		Taxonomic group	2	31.00	< 0.001
		Predictors: taxonomic group	22	3.44	< 0.001
	Predictors + range size + predictors: range size	Predictors	11	47.36	< 0.001
		Range size	2	17.58	< 0.001
		Predictors: range size	22	6.68	< 0.001

Predictors comprised 11 land use and land-use intensity classes. A colon represents an interaction between two model variables. Only species with the most stable predictor coefficient estimates (0 ≤ species’ mean coefficient of variation of predictor coefficients ≤ 1.06, as measured with cross-validation) were included (*n* = 166)

Overall, the relative suitability of different land-use and land-use-intensity classes for cropland and forest species differed significantly among taxonomic groups. However, the effect of land-use intensity on habitat suitability was only significant for cropland birds, for whom high-intensity forestry was significantly more suitable than low-intensity forestry (Fig. 2). Nevertheless, low-intensity cropland and grassland were more suitable than the equivalent land uses of high intensity for cropland birds, whereas the opposite was true for cropland mammals. On the other hand, land-use intensity did not significantly affect the suitability of any land use for any taxonomic group of forest species.

Overall, the relative suitability of different land-use and land-use-intensity classes for cropland and forest species differed significantly among range-size groups. Urban was the most suitable land use for large-range-sized cropland and forest species. However, low-intensity grassland was the most suitable land use for small-range-sized cropland species, and low-intensity forestry was the most suitable land use for small-range-sized forest species. For large- and medium-range-sized cropland species, forestry intensification had a significant positive effect on habitat suitability. For large-range-sized forest species, cropland, grassland and forestry intensification all had significant positive effects on habitat suitability. On the other hand, low-intensity grassland was significantly more suitable than high-intensity grassland for medium-range-sized cropland species.

The mean AUC of all 229 species was 0.62, while for the 166 species with the most robust coefficient estimates, mean AUC was 0.60, neither of which would typically indicate good model discriminatory capacity (see Online Resource 6). However, our aim was not to generate accurate predictions of species distributions according to existing occurrence records, but to assess the identifiability and relative effects of land-use and land-use intensity variables on habitat suitability. We therefore report AUC values for informative purposes only.

## Discussion

Understanding the effects of land use and land-use intensity on biodiversity at different spatial scales is crucial for conservation planning (Duncan et al. 2020; Pearman-Gillman et al. 2020). However, few studies have used SDMs to assess how land-use and land-use intensity variables affect habitat suitability for individual vertebrate species on broad spatial scales. Here, we used a novel SDM approach to investigate land use and land-use-intensity effects on habitat suitability for vertebrates throughout their contemporary ranges across Europe, summarising the effects for different species groups on a regional scale. For 166 cropland and forest species, we identified significant effects of land use and land-use intensity on habitat suitability. We found variability in the effects among species groups, including groups based on range size, providing important insights into the relationship between land management and the occurrence of different species. Our approach was innovative in several key ways: the application of land-use intensity data to SDMs, the integration of ecologically-informed priors based on expert knowledge of species-ecosystem associations to derive more ecologically realistic response functions, the identification of unsuitable land uses for pseudoabsence sampling, the focus on the stability and robustness of model coefficients to inform model performance (in lieu of traditional validation metrics), and the assessment of regional-scale effects of land use and land-use intensity. Compared to the more commonly applied approach of summarising local-scale effects across a broader area (Newbold et al. 2015; Beckmann et al. 2019), our prior-informed SDM approach explicitly accounts for broad-scale (range-wide) effects on biodiversity, enabling new insights into how land use influences biodiversity on broad spatial scales.

Previous research has shown that land-system variables can be important drivers of vertebrate habitat suitability and species distributions, including land-use (Cardador and Blackburn 2019; Pearman-Gillman et al. 2020; Devenish et al. 2021) and land-use-intensity variables (Duncan et al. 2020; O'Connor et al. 2024). Although we found that our set of 11 land-use and land-use-intensity variables had a significant effect on vertebrate habitat suitability, significant differences in habitat suitability due to land-use intensity of the same land use were relatively rare. Therefore, while we could identify landscape-scale land-use and land-use intensity as important factors influencing habitat suitability, our results suggested that – overall – land use generally led to greater differences in habitat suitability than land-use intensity. However, we found that the effects of land-use intensity were greater for certain species groups; notably, cropland birds, medium- to large-range-sized cropland species and large-range-sized forest species. Thus, our findings support previous studies which also found that geographic range size was an important determinant of species’ responses to land-use change and intensification (Newbold et al. 2018; Etard and Newbold 2024).

In cropland systems, intensification has resulted in increased mechanisation, enhanced suppression of non-target vegetation with chemicals, increased size and homogeneity of individual farms and decreased habitat and landscape heterogeneity (Van Der Sluis et al. 2016; Maskell et al. 2019; Dullinger et al. 2021). The loss of landscape-scale habitat diversity that is associated with intensive cropland systems has been shown to negatively affect cropland bird diversity (Bosco et al., 2024). Studies using SDMs have also found negative correlations between cropland or agricultural area and mammal habitat suitability (Devenish et al. 2021; Davoli et al. 2024). In line with previous research, we found that the occurrence of cropland vertebrates was negatively associated with cropland intensification on a landscape scale (although the difference was not statistically significant). Furthermore, we found that cropland birds were more sensitive to cropland and grassland use-intensity than cropland mammals, perhaps supporting previous indications that cropland mammals could be more resilient to agricultural intensification than cropland birds (Šálek et al. 2018).

In forestry systems, variables such as rotation length, tree species composition and type of forest regeneration are important factors relating to intensification (Duncker et al. 2012; Schall and Ammer 2013) and habitat suitability. While there is limited SDM-based research on the effects of forestry intensification, Hofner et al. (2011) showed that bird SDM partial response curves could differ depending on forest type (e.g. coniferous v. broadleaf). Overall, we found little effect of forestry intensification on vertebrate habitat suitability, in line with previous findings that forestry intensification had limited effect on carnivorous mammal habitat use (Jones et al. 2024), forest bird richness (Sabatini et al. 2016; Asbeck et al. 2021) and bat richness (Asbeck et al. 2021). However, forestry intensification can cause changes in species composition, to the detriment of rare (Leso et al. 2019) or threatened (Jones et al. 2024) species. For example, shorter rotation periods, indicative of more intensive management, can decrease habitat suitability for undisturbed forest specialist species, while encouraging other, non-specialist species (Van 'T Veen et al. 2025). We did find evidence for some negative – albeit statistically insignificant – effects of forestry intensification on small-range-sized vertebrates. Forestry intensification also had significant positive effects on habitat suitability for several other species groups, particularly large-range-sized species. These results thus support previous findings that vertebrates in general may be more resilient to forestry intensification than to agricultural intensification (Beckmann et al. 2019), while suggesting that this pattern may be particularly evident for wider-ranging species.

Previous SDM-based studies have shown that urban land area (Hofner et al. 2011) and urban land-use intensity can negatively affect vertebrate habitat suitability (Duncan et al. 2020), with human population density having been identified as a major driver of large mammal habitat loss in Europe (Davoli et al. 2024). Other research has also found that human land uses typically have more wide-ranging, generalist species, and fewer narrow-ranged, specialist species than natural vegetation (Slatyer et al. 2013; Newbold et al. 2018). Indeed, we found that the difference in habitat suitability between small- and large-range-sized species was greatest in urban landscapes, with urban land uses particularly benefitting wide-ranging species. Conversion of more suitable habitats (e.g. cropland or low-intensity grassland for cropland species or forest for forest species) to urban areas could therefore have a negative effect on habitat suitability for more specialised species, but a positive effect for more widespread species. Similarly, we found that land-use intensification generally favoured species with larger ranges, which were less likely than narrow-ranged species to be threatened with extinction, in line with previous research (Newbold et al. 2018). These findings therefore indicate that land-use intensification can affect community structure by disproportionately reducing habitat suitability for narrow‑ranged species. The loss of rarer, narrow-ranged species – that have more specific climatic niches, distinct traits and unique functional roles – can have negative consequences for ecosystem functioning as well as biodiversity conservation (Mouillot et al. 2013). At the same time, the beneficial effects of land-use intensification on wide-ranging species highlight that common, generalist species can be important drivers of regional biodiversity patterns (Wayman et al. 2024).

Over the coming decades, increased demand for agricultural and forestry products for the bioeconomy, alongside terrestrial conservation and climate-mitigation measures, are likely to cause land-use and land-use intensity changes (Dou et al. 2023; Visconti et al. 2024). However, there is considerable uncertainty in land-use projections (Alexander et al. 2017) and – as we have shown – different species groups can respond differently to the same changes. Therefore, having a flexible approach to predicting the potential effects of future land-use changes on biodiversity – which can be applied to different regions or taxonomic groups – is useful for guiding land-use planning and supporting decisions about conservation, management, agriculture and forestry (Urban et al. 2021). Our prior-informed SDMs can also be used to guide conservation actions for specific groups. For example, our results suggest that reaching the target of increasing farmland bird populations in the EU (Regulation (EU) 2024/1991) could be facilitated by reducing the intensity of cropland and grassland management, or by increasing the availability of water and wetland habitats on a landscape scale (see also Chapman et al. 2025). Furthermore, our results suggest that reducing forestry intensity could be beneficial for rarer, more specialised forest species, but less so for more common forest species.

Here, we used prior-informed SDMs to provide a regional-scale, multi-taxa summary of the effects of land use and land-use intensity on a specific group of vertebrates, within their contemporary ranges. We prioritised identifying robust statistical relationships between species occurrence rates and SDM predictors, rather than on aligning model spatial predictions with existing occurrence records. As a result, our study included models with poor discriminatory capacity according to AUC. Nevertheless, known limitations of traditional SDM validation metrics, including AUC, mean that alternative approaches to evaluating model performance are required (Lobo et al. 2008; Fourcade et al. 2018). Thus, our approach addressed a current challenge in SDM evaluation. We also prioritised the integration of expert ecological knowledge into our SDMs to increase the realism of the estimated effects of land use and land-use intensity. Therefore, our approach could be used in other studies with similar aims. Nevertheless, our results rely on the accuracy of the expert knowledge used, and on the modelling decisions made, and further testing and validation of our approach is necessary.

### Limitations

Our models were subject to the same limitations and biases common to all large-scale biodiversity syntheses, only reflecting patterns supported by the available data (Hughes et al. 2021). For example, the biodiversity records may have been subject to sampling biases (although we did not find evidence of particular bias e.g. towards urban areas). Similarly, species ranges may have been oversimplified (Herkt et al. 2017) or temporally outdated (Henry et al. 2024); however, we combined estimates from multiple sources, wherever possible, making our estimates conservative. Our approach used static biodiversity and land-use data and did not account for the potential effects of time lags on biodiversity (Essl et al. 2024). Furthermore, to improve model interpretability, we did not account for the effects on biodiversity of climate (Newbold et al. 2018; Festa et al. 2023), interactions between land uses (Outhwaite et al. 2022), landscape connectivity (Gil-Tena et al. 2014), or other landscape-configuration variables (Duflot et al. 2017).

### Further research

Improvements to SDM predictive performance, especially for generalist species, could be made by using binary land-use predictors (Gábor et al. 2024) at a finer thematic (Marshall et al. 2021) or spatial resolution (Connor et al. 2018), or by using independent training and testing datasets rather than spatial block cross-validation (Roberts et al. 2017; Fourcade et al. 2018). The use of other SDM methods – for example those allowing for more complex relationships – could further improve model predictive performance, provide more accurate partial effects for some species (Valavi et al. 2022), and enable identification of threshold responses to increasing landscape shares of different land uses, which may be more informative than linear responses (Melo et al. 2018). However, the importance of coefficient priors in such models, and the form such priors should take, requires further exploration. Additionally, to our knowledge, a generic framework for the validation of coefficients, rather than spatial predictions, does not yet exist.

Although beyond the scope of this study, inclusion of priors that account for species’ responses to land-use intensity, in addition to land use, could improve the realism of the results. More detailed investigations into the effects of different aspects of land-use intensity could provide insights into the mechanisms behind species’ responses to land-use intensity. Additionally, the analysis could be expanded to include urban-, grassland- and wetland species, and further research could explore how the changes in biodiversity identified affect ecosystem functioning and resilience on a regional scale. Lastly, we prioritised the estimation of realistic, interpretable and robust relationships between land use and land-use intensity and species habitat suitability over model spatial predictive performance. However, further research should explore alternative approaches that can better balance statistical robustness, ecological realism, interpretability and model predictive performance.

## Conclusion

Land use and land-use intensity are key drivers of global biodiversity change. However, in contrast to climate change, few studies have used SDMs to estimate the effects of land-use and land-use intensity on biodiversity on broad spatial scales, instead summarising local-scale biodiversity effects. Here, we developed a novel SDM-based approach that can be used to estimate the effects of land use and land-use intensity on biodiversity on broad spatial scales, taking into account expert ecological knowledge. Based on our assessment of cropland and forest vertebrates across Europe, we show that land use and land-use intensity affect species’ habitat suitability and biodiversity patterns on broad spatial scales, with likely implications for the structure of ecological communities, biodiversity conservation and ecosystem functioning.

## Supplementary Information

Below is the link to the electronic supplementary material.

Supplementary file1 (DOCX 389 KB)

## Data Availability

The data used in this study are available from the sources detailed in the Methods section.
