# Supplementary material for: Regional land use and land-use intensity effects on vertebrate biodiversity across Europe identified using species distribution models
Source: Landsc Ecol. 2026 May 18;41(7):122. doi: 10.1007/s10980-026-02379-y (PMC13364868; doi:10.1007/s10980-026-02379-y)
Supplement: Supplementary file 1 — Supplementary file1 (DOCX 389 KB) [file 10980_2026_2379_MOESM1_ESM.docx]

Regional land use and land-use intensity effects on vertebrate biodiversity across Europe identified using species distribution models

**Supplementary Material**

1. Further methodological details.

Land-use intensity

Cropland use-intensity was based on field size and nitrogen application, while grassland use-intensity was based on nitrogen application, livestock density and mowing frequency (Dou et al., 2021). Forest use-intensity was based on forest management classes from Oostdijk et al. (2023). As such, low-intensity forestry included primary forest with barely any management, close-to-nature forest that had been previously disturbed and received management to enhance ecosystem functioning, and combined-objective forestry, where management may have been for protection, wood production, recreation or other purposes. High-intensity forestry included forests that were managed intensively and predominantly for wood production, which could have had large areas of forest cut down over short or longer rotation cycles, and which may have consisted of fast-growing species only. Urban use-intensity was based on imperviousness, as given by European Union's Copernicus Land Monitoring Service (2018).

Species distribution model evaluation

The area under the receiver operating characteristic curve (AUC) measures how well a model can discriminate between presences and absences in the data used; however, relying on AUC to evaluate model performance is questionable when true absences are unavailable, and when the aim is not to estimate the realised distribution of a species (Jiménez-Valverde, 2012). Furthermore, validation of the predictions of a model based on similarly biased data may not necessarily reflect the quality of such predictions, or the alignment of the predictions with expert knowledge. Thus, due to its focus on how well a model predicts species presences and absences, rather than on how well the predictors explain occurrences, AUC has been criticised as ignoring the goodness-of-fit of models and overrating ecologically meaningless SDMs (Lobo et al., 2008, Jiménez-Valverde, 2012, Fourcade et al., 2018). Further issues with AUC exist in relation to the effects of sample size (van Proosdij et al., 2016), geographic extent (Lobo et al., 2008), and the inability to effectively make comparisons between species (Jiménez-Valverde, 2012).

1. Species list. IUCN Red List Category results are for European Regional assessments (IUCN, 2024).

| **Scientific name** | **Common name** | **Taxonomic group** | **IUCN Red List Category** |
| --- | --- | --- | --- |
| *Accipiter brevipes* | Levant sparrowhawk | Birds | Least concern |
| *Accipiter gentilis* | Northern goshawk | Birds | Least concern |
| *Acrocephalus dumetorum* | Blyth’s reed-warbler | Birds | Least concern |
| *Aegolius funereus* | Boreal owl | Birds | Least concern |
| *Aegypius monachus* | Cinereous vulture | Birds | Least concern |
| *Alces alces* | Moose | Mammals | Not available |
| *Algyroides nigropunctatus* | Blue-throated keeled lizard | Herpetofauna | Least concern |
| *Alytes obstetricans* | Common midwife toad | Herpetofauna | Least concern |
| *Anser anser* | Greylag goose | Birds | Least concern |
| *Anser erythropus* | Lesser white-fronted goose | Birds | Vulnerable |
| *Anser fabalis* | Bean goose | Birds | Least concern |
| *Apodemus agrarius* | Striped field mouse | Mammals | Least concern |
| *Apodemus flavicollis* | Yellow-necked field mouse | Mammals | Least concern |
| *Apodemus sylvaticus* | Long-tailed field mouse | Mammals | Least concern |
| *Aquila clanga* | Greater spotted eagle | Birds | Vulnerable |
| *Aquila heliaca* | Eastern imperial eagle | Birds | Least concern |
| *Aquila pomarina* | Lesser spotted eagle | Birds | Least concern |
| *Asio flammeus* | Short-eared owl | Birds | Least concern |
| *Asio otus* | Long-eared owl | Birds | Least concern |
| *Athene noctua* | Little owl | Birds | Least concern |
| *Barbastella barbastellus* | Barbastelle bat | Mammals | Vulnerable |
| *Bison bonasus* | European bison | Mammals | Vulnerable |
| *Bombina bombina* | Fire-bellied toad | Herpetofauna | Least concern |
| *Bombina variegata* | Yellow-bellied toad | Herpetofauna | Least concern |
| *Bombycilla garrulus* | Bohemian waxwing | Birds | Least concern |
| *Branta leucopsis* | Barnacle goose | Birds | Least concern |
| *Bubo bubo* | Eurasian eagle-owl | Birds | Least concern |
| *Bucephala clangula* | Common goldeneye | Birds | Least concern |
| *Bufo bufo* | Common toad | Herpetofauna | Least concern |
| *Buteo lagopus* | Rough-legged buzzard | Birds | Least concern |
| *Calandrella brachydactyla* | Greater short-toed lark | Birds | Least concern |
| *Canis lupus* | Grey wolf | Mammals | Least concern |
| *Capreolus capreolus* | European roe deer | Mammals | Least concern |
| *Caprimulgus europaeus* | European nightjar | Birds | Least concern |
| *Caprimulgus ruficollis* | Red-necked nightjar | Birds | Near threatened |
| *Carduelis citrinella* | Citril finch | Birds | Least concern |
| *Castor fiber* | Eurasian beaver | Mammals | Least concern |
| *Certhia familiaris* | Eurasian treecreeper | Birds | Least concern |
| *Chalcides striatus* | Western three-toed skink | Herpetofauna | Least concern |
| *Chioglossa lusitanica* | Golden-striped salamander | Herpetofauna | Near threatened |
| *Ciconia nigra* | Black stork | Birds | Least concern |
| *Circaetus gallicus* | Short-toed snake-eagle | Birds | Least concern |
| *Circus macrourus* | Pallid harrier | Birds | Least concern |
| *Circus pygargus* | Montagu’s harrier | Birds | Least concern |
| *Cisticola juncidis* | Zitting cisticola | Birds | Least concern |
| *Coccothraustes coccothraustes* | Hawfinch | Birds | Least concern |
| *Colinus virginianus* | Northern bobwhite | Birds | Not available |
| *Coracias garrulus* | European roller | Birds | Least concern |
| *Corvus frugilegus* | Rook | Birds | Vulnerable |
| *Coturnix coturnix* | Common quail | Birds | Near threatened |
| *Crex crex* | Corncrake | Birds | Least concern |
| *Cricetus cricetus* | European hamster | Mammals | Critically endangered |
| *Crocidura leucodon* | Bicoloured shrew | Mammals | Least concern |
| *Crocidura suaveolens* | Lesser white-toothed shrew | Mammals | Least concern |
| *Cygnus cygnus* | Whopper swan | Birds | Least concern |
| *Dama dama* | Common fallow deer | Mammals | Least concern |
| *Dendrocopos leucotos* | White-backed woodpecker | Birds | Least concern |
| *Dendrocopos syriacus* | Syrian woodpecker | Birds | Least concern |
| *Dryocopus martius* | Black woodpecker | Birds | Least concern |
| *Dryomys nitedula* | Forest dormouse | Mammals | Least concern |
| *Elaphe sauromates* | Eastern four-lined ratsnake | Herpetofauna | Least concern |
| *Eliomys quercinus* | Garden dormouse | Mammals | Vulnerable |
| *Emberiza aureola* | Yellow-breasted bunting | Birds | Critically endangered |
| *Emberiza cirlus* | Cirl bunting | Birds | Least concern |
| *Emberiza hortulana* | Ortolan bunting | Birds | Least concern |
| *Emberiza melanocephala* | Black-headed bunting | Birds | Least concern |
| *Emberiza pusilla* | Little bunting | Birds | Least concern |
| *Emberiza rustica* | Rustic bunting | Birds | Least concern |
| *Eptesicus nilssonii* | Northern bat | Mammals | Least concern |
| *Eptesicus serotinus* | Serotine bat | Mammals | Least concern |
| *Erinaceus concolor* | Southern white-breasted hedgehog | Mammals | Not applicable |
| *Erinaceus europaeus* | Western European hedgehog | Mammals | Near threatened |
| *Erythropygia galactotes* | Rufous-tailed Scrub-robin | Birds | Least concern |
| *Falco columbarius* | Merlin | Birds | Vulnerable |
| *Falco subbuteo* | Eurasian hobby | Birds | Least concern |
| *Falco vespertinus* | Red-footed falcon | Birds | Vulnerable |
| *Felis silvestris* | European wildcat | Mammals | Not available |
| *Ficedula albicollis* | Collared flycatcher | Birds | Least concern |
| *Ficedula hypoleuca* | European pied flycatcher | Birds | Least concern |
| *Ficedula parva* | Red-breasted flycatcher | Birds | Least concern |
| *Ficedula semitorquata* | Semi-collared flycatcher | Birds | Least concern |
| *Fringilla montifringilla* | Brambling | Birds | Least concern |
| *Galerida cristata* | Crested lark | Birds | Least concern |
| *Genetta genetta* | Common genet | Mammals | Least concern |
| *Glaucidium passerinum* | Eurasian pygmy-owl | Birds | Least concern |
| *Grus grus* | Common crane | Birds | Least concern |
| *Gulo gulo* | Wolverine | Mammals | Vulnerable |
| *Haliaeetus albicilla* | White-tailed sea-eagle | Birds | Least concern |
| *Herpestes ichneumon* | Egyptian mongoose | Mammals | Least concern |
| *Hieraaetus pennatus* | Booted eagle | Birds | Least concern |
| *Hippolais icterina* | Icterine warbler | Birds | Least concern |
| *Hippolais olivetorum* | Olive-tree warbler | Birds | Least concern |
| *Hippolais polyglotta* | Melodious warbler | Birds | Least concern |
| *Jynx torquilla* | Eurasian wryneck | Birds | Least concern |
| *Lagopus lagopus* | Willow grouse | Birds | Least concern |
| *Lanius excubitor* | Great grey shrike | Birds | Least concern |
| *Lanius minor* | Lesser grey shrike | Birds | Least concern |
| *Lanius nubicus* | Masked shrike | Birds | Least concern |
| *Lanius senator* | Woodchat shrike | Birds | Near threatened |
| *Lepus europaeus* | European hare | Mammals | Least concern |
| *Lepus granatensis* | Iberian hare | Mammals | Least concern |
| *Lepus timidus* | Mountain hare | Mammals | Least concern |
| *Loxia curvirostra* | Red crossbill | Birds | Least concern |
| *Loxia leucoptera* | Two-barred crossbill | Birds | Least concern |
| *Loxia pytyopsittacus* | Parrot crossbill | Birds | Least concern |
| *Loxia scotica* | Scottish crossbill | Birds | Least concern |
| *Lullula arborea* | Woodlark | Birds | Least concern |
| *Luscinia luscinia* | Thrush nightingale | Birds | Least concern |
| *Lynx lynx* | Eurasian lynx | Mammals | Least concern |
| *Lynx pardinus* | Iberian lynx | Mammals | Vulnerable |
| *Martes foina* | Beech marten | Mammals | Least concern |
| *Martes martes* | Pine marten | Mammals | Least concern |
| *Melanocorypha calandra* | Calandra lark | Birds | Least concern |
| *Meleagris gallopavo* | Wild turkey | Birds | Not available |
| *Meles meles* | Eurasian badger | Mammals | Least concern |
| *Micromys minutus* | European harvest mouse | Mammals | Least concern |
| *Microtus agrestis* | Short-tailed field vole | Mammals | Least concern |
| *Microtus arvalis* | Common vole | Mammals | Least concern |
| *Microtus duodecimcostatus* | Mediterranean pine vole | Mammals | Least concern |
| *Microtus gerbei* | Pyrenean pine vole | Mammals | Least concern |
| *Microtus lusitanicus* | Lusitanian pine vole | Mammals | Least concern |
| *Microtus subterraneus* | European pine vole | Mammals | Least concern |
| *Milvus migrans* | Black kite | Birds | Least concern |
| *Milvus milvus* | Red kite | Birds | Least concern |
| *Miniopterus schreibersii* | Schreiber's bat | Mammals | Vulnerable |
| *Muntiacus reevesi* | Reeves’ muntjac | Mammals | Not available |
| *Mus musculus* | House mouse | Mammals | Least concern |
| *Mus spretus* | Western Mediterranean mouse | Mammals | Least concern |
| *Muscardinus avellanarius* | Hazel dormouse | Mammals | Least concern |
| *Mustela erminea* | Stoat | Mammals | Least concern |
| *Mustela putorius* | European polecat | Mammals | Least concern |
| *Myopus schisticolor* | Wood lemming | Mammals | Least concern |
| *Myotis alcathoe* | Alcathoe's bat | Mammals | Near threatened |
| *Myotis bechsteinii* | Bechstein's bat | Mammals | Vulnerable |
| *Myotis brandtii* | Brandt's bat | Mammals | Least concern |
| *Myotis capaccinii* | Long-fingered bat | Mammals | Vulnerable |
| *Myotis dasycneme* | Pond bat | Mammals | Vulnerable |
| *Myotis daubentonii* | Daubenton’s bat | Mammals | Least concern |
| *Myotis emarginatus* | Geoffroy’s bat | Mammals | Least concern |
| *Myotis myotis* | Greater mouse-eared bat | Mammals | Least concern |
| *Nucifraga caryocatactes* | Northern nutcracker | Birds | Least concern |
| *Numenius arquata* | Eurasian curlew | Birds | Near threatened |
| *Numenius phaeopus* | Whimbrel | Birds | Least concern |
| *Numida meleagris* | Helmeted guineafowl | Birds | Not available |
| *Nyctalus lasiopterus* | Greater noctule bat | Mammals | Vulnerable |
| *Nyctalus leisleri* | Leisler's bat | Mammals | Least concern |
| *Nyctereutes procyonoides* | Raccoon dog | Mammals | Not available |
| *Nycticorax nycticorax* | Black-crowned night heron | Birds | Least concern |
| *Oriolus oriolus* | Eurasian golden oriole | Birds | Least concern |
| *Oryctolagus cuniculus* | European rabbit | Mammals | Near threatened |
| *Otis tarda* | Great bustard | Birds | Least concern |
| *Otus scops* | Cyprus scops-owl | Birds | Least concern |
| *Pandion haliaetus* | Osprey | Birds | Least concern |
| *Passer hispaniolensis* | Spanish sparrow | Birds | Least concern |
| *Pavo cristatus* | Indian peafowl | Birds | Not available |
| *Perdix perdix* | Grey partridge | Birds | Least concern |
| *Perisoreus infaustus* | Siberian jay | Birds | Least concern |
| *Phylloscopus bonelli* | Western Bonelli’s warbler | Birds | Least concern |
| *Phylloscopus borealis* | Arctic warbler | Birds | Least concern |
| *Phylloscopus ibericus* | Iberian chiffchaff | Birds | Least concern |
| *Phylloscopus trochiloides* | Greenish warbler | Birds | Least concern |
| *Picoides tridactylus* | Three-toed woodpecker | Birds | Least concern |
| *Picus canus* | Grey-faced woodpecker | Birds | Least concern |
| *Pinicola enucleator* | Pine grosbeak | Birds | Least concern |
| *Pipistrellus kuhlii* | Kuhl’s pipistrelle | Mammals | Least concern |
| *Pipistrellus nathusii* | Nathusius’ pipistrelle | Mammals | Least concern |
| *Pipistrellus pipistrellus* | Common pipistrelle | Mammals | Least concern |
| *Pipistrellus pygmaeus* | Soprano pipistrelle | Mammals | Least concern |
| *Plecotus auritus* | Brown long-eared bat | Mammals | Least concern |
| *Pluvialis apricaria* | Eurasian golden plover | Birds | Least concern |
| *Podarcis bocagei* | Bocage’s wall lizard | Herpetofauna | Least concern |
| *Pteromys volans* | Siberian flying squirrel | Mammals | Near threatened |
| *Pyrrhula murina* | Azores bullfinch | Birds | Vulnerable |
| *Rana arvalis* | Moor frog | Herpetofauna | Least concern |
| *Rana dalmatina* | Agile frog | Herpetofauna | Least concern |
| *Rana graeca* | Greek stream frog | Herpetofauna | Least concern |
| *Rana temporaria* | European common frog | Herpetofauna | Least concern |
| *Rangifer tarandus* | Reindeer | Mammals | Least concern |
| *Rattus rattus* | House rat | Mammals | Least concern |
| *Rhinolophus euryale* | Mediterranean horseshoe bat | Mammals | Vulnerable |
| *Rhinolophus ferrumequinum* | Greater horseshoe bat | Mammals | Least concern |
| *Rhinolophus hipposideros* | Lesser horseshoe bat | Mammals | Least concern |
| *Rhinolophus mehelyi* | Mehely's horseshoe bat | Mammals | Endangered |
| *Salamandra atra* | Alpine salamander | Herpetofauna | Least concern |
| *Salamandra salamandra* | Common fire salamander | Herpetofauna | Vulnerable |
| *Sciurus anomalus* | Caucasian squirrel | Mammals | Not applicable |
| *Sciurus vulgaris* | Eurasian red squirrel | Mammals | Least concern |
| *Scolopax rusticola* | Eurasian woodcock | Birds | Least concern |
| *Sicista betulina* | Northern birch mouse | Mammals | Least concern |
| *Sitta krueperi* | Krueper’s nuthatch | Birds | Least concern |
| *Sitta whiteheadi* | Corsican nuthatch | Birds | Vulnerable |
| *Sorex araneus* | Common shrew | Mammals | Least concern |
| *Sorex coronatus* | Crowned shrew | Mammals | Least concern |
| *Sorex minutus* | Eurasian pygmy shrew | Mammals | Least concern |
| *Spermophilus citellus* | European ground squirrel | Mammals | Endangered |
| *Streptopelia turtur* | European turtle-dove | Birds | Vulnerable |
| *Strix aluco* | Tawny owl | Birds | Least concern |
| *Strix nebulosa* | Great grey owl | Birds | Least concern |
| *Strix uralensis* | Ural owl | Birds | Least concern |
| *Sturnus unicolor* | Spotless starling | Birds | Least concern |
| *Suncus etruscus* | Pygmy white-toothed shrew | Mammals | Least concern |
| *Surnia ulula* | Northern hawk-owl | Birds | Least concern |
| *Sus scrofa* | Wild boar | Mammals | Least concern |
| *Sylvia curruca* | Lesser whitethroat | Birds | Least concern |
| *Sylvia hortensis* | Western Orphean warbler | Birds | Least concern |
| *Sylvia melanocephala* | Sardinian warbler | Birds | Least concern |
| *Sylvia nisoria* | Barred warbler | Birds | Least concern |
| *Syrmaticus reevesii* | Reeves’s pheasant | Birds | Not available |
| *Tadarida teniotis* | European free-tailed bat | Mammals | Least concern |
| *Talpa europaea* | European mole | Mammals | Least concern |
| *Talpa occidentalis* | Iberian mole | Mammals | Least concern |
| *Tamias sibiricus* | Siberian chipmunk | Mammals | Least concern |
| *Tarsiger cyanurus* | Orange-flanked bush-robin | Birds | Least concern |
| *Testudo hermanni* | Hermann’s tortoise | Herpetofauna | Vulnerable |
| *Tetrao urogallus* | Western capercaillie | Birds | Least concern |
| *Tetrax tetrax* | Little bustard | Birds | Vulnerable |
| *Tringa ochropus* | Green sandpiper | Birds | Least concern |
| *Triturus carnifex* | Italian crested newt | Herpetofauna | Vulnerable |
| *Triturus cristatus* | Great crested newt | Herpetofauna | Least concern |
| *Triturus dobrogicus* | Danube crested newt | Herpetofauna | Least concern |
| *Triturus marmoratus* | Marbled newt | Herpetofauna | Vulnerable |
| *Turdus iliacus* | Redwing | Birds | Least concern |
| *Turdus pilaris* | Fieldfare | Birds | Least concern |
| *Turdus torquatus* | Ring ouzel | Birds | Least concern |
| *Tyto alba* | Common barn-owl | Birds | Least concern |
| *Upupa epops* | Common hoopoe | Birds | Least concern |
| *Ursus arctos* | Brown bear | Mammals | Least concern |
| *Vespertilio murinus* | Parti-coloured bat | Mammals | Least concern |
| *Vulpes vulpes* | Red fox | Mammals | Least concern |

1. List of species-land-use suitability. A “yes” means suitable, while a “no” means unsuitable. Species-land-use suitability was based on species-ecosystem associations from Roscher et al. (2015).

| **Species name** | **Land-use suitability** | | | | | |
| --- | --- | --- | --- | --- | --- | --- |
|  | **Cropland** | **Forest** | **Grassland** | **Water & wetland** | **Bare, rock & shrub** | **Urban** |
| *Accipiter brevipes* | Yes | Yes | No | No | No | No |
| *Accipiter gentilis* | No | Yes | No | No | No | No |
| *Acrocephalus dumetorum* | No | Yes | No | Yes | No | No |
| *Aegolius funereus* | No | Yes | No | No | No | No |
| *Aegypius monachus* | No | Yes | No | No | Yes | No |
| *Alces alces* | Yes | Yes | Yes | Yes | No | No |
| *Algyroides nigropunctatus* | Yes | Yes | No | Yes | Yes | Yes |
| *Alytes obstetricans* | Yes | Yes | Yes | Yes | Yes | Yes |
| *Anser anser* | Yes | No | Yes | Yes | Yes | No |
| *Anser erythropus* | Yes | No | Yes | Yes | No | No |
| *Anser fabalis* | Yes | No | Yes | Yes | No | No |
| *Apodemus agrarius* | Yes | Yes | Yes | Yes | No | Yes |
| *Apodemus flavicollis* | Yes | Yes | Yes | No | Yes | Yes |
| *Apodemus sylvaticus* | Yes | Yes | Yes | Yes | No | Yes |
| *Aquila clanga* | No | Yes | No | Yes | No | No |
| *Aquila heliaca* | No | Yes | Yes | No | No | No |
| *Aquila pomarina* | No | Yes | Yes | No | No | No |
| *Asio flammeus* | Yes | No | Yes | Yes | No | No |
| *Asio otus* | Yes | Yes | No | No | No | No |
| *Athene noctua* | Yes | Yes | Yes | No | No | No |
| *Barbastella barbastellus* | Yes | Yes | Yes | Yes | Yes | Yes |
| *Bison bonasus* | No | Yes | Yes | No | No | No |
| *Bombina bombina* | Yes | Yes | Yes | Yes | Yes | No |
| *Bombina variegata* | Yes | Yes | Yes | Yes | Yes | No |
| *Bombycilla garrulus* | No | Yes | No | No | No | No |
| *Branta leucopsis* | Yes | No | Yes | Yes | Yes | Yes |
| *Bubo bubo* | No | Yes | No | No | Yes | No |
| *Bucephala clangula* | No | Yes | No | Yes | No | No |
| *Bufo bufo* | Yes | Yes | Yes | Yes | Yes | Yes |
| *Buteo lagopus* | Yes | Yes | Yes | No | Yes | No |
| *Calandrella brachydactyla* | Yes | No | Yes | No | No | No |
| *Canis lupus* | Yes | Yes | Yes | Yes | Yes | Yes |
| *Capreolus capreolus* | Yes | Yes | Yes | Yes | No | Yes |
| *Caprimulgus europaeus* | No | Yes | No | No | No | No |
| *Caprimulgus ruficollis* | No | Yes | No | No | No | No |
| *Carduelis citrinella* | No | Yes | No | No | No | No |
| *Castor fiber* | Yes | Yes | Yes | Yes | No | Yes |
| *Certhia familiaris* | No | Yes | No | No | No | No |
| *Chalcides striatus* | No | Yes | Yes | No | No | Yes |
| *Chioglossa lusitanica* | Yes | Yes | Yes | Yes | Yes | No |
| *Ciconia nigra* | No | Yes | No | Yes | No | No |
| *Circaetus gallicus* | No | Yes | Yes | No | No | No |
| *Circus macrourus* | Yes | No | Yes | No | No | No |
| *Circus pygargus* | Yes | No | Yes | No | No | No |
| *Cisticola juncidis* | Yes | No | Yes | No | No | No |
| *Coccothraustes coccothraustes* | Yes | Yes | No | No | No | No |
| *Colinus virginianus* | Yes | No | No | No | No | No |
| *Coracias garrulus* | Yes | Yes | No | No | No | No |
| *Corvus frugilegus* | Yes | No | No | No | No | Yes |
| *Coturnix coturnix* | Yes | No | Yes | No | No | No |
| *Crex crex* | Yes | No | Yes | Yes | No | No |
| *Cricetus cricetus* | Yes | No | Yes | No | No | Yes |
| *Crocidura leucodon* | Yes | Yes | Yes | Yes | Yes | Yes |
| *Crocidura suaveolens* | Yes | Yes | Yes | Yes | Yes | Yes |
| *Cygnus cygnus* | Yes | No | Yes | Yes | No | No |
| *Dama dama* | Yes | Yes | Yes | Yes | No | Yes |
| *Dendrocopos leucotos* | No | Yes | No | No | No | No |
| *Dendrocopos syriacus* | Yes | Yes | No | No | No | Yes |
| *Dryocopus martius* | No | Yes | No | No | No | No |
| *Dryomys nitedula* | Yes | Yes | Yes | No | Yes | No |
| *Elaphe sauromates* | Yes | Yes | Yes | Yes | Yes | No |
| *Eliomys quercinus* | Yes | Yes | No | No | Yes | Yes |
| *Emberiza aureola* | Yes | Yes | No | No | No | No |
| *Emberiza cirlus* | Yes | No | Yes | No | No | No |
| *Emberiza hortulana* | Yes | No | Yes | No | No | No |
| *Emberiza melanocephala* | Yes | No | No | No | Yes | No |
| *Emberiza pusilla* | No | Yes | No | Yes | No | No |
| *Emberiza rustica* | No | Yes | No | Yes | No | No |
| *Eptesicus nilssonii* | Yes | Yes | Yes | Yes | Yes | Yes |
| *Eptesicus serotinus* | Yes | Yes | Yes | Yes | Yes | Yes |
| *Erinaceus concolor* | Yes | Yes | Yes | Yes | Yes | Yes |
| *Erinaceus europaeus* | Yes | Yes | Yes | No | Yes | Yes |
| *Erythropygia galactotes* | Yes | No | No | No | No | No |
| *Falco columbarius* | No | Yes | Yes | Yes | Yes | No |
| *Falco subbuteo* | Yes | Yes | No | Yes | No | No |
| *Falco vespertinus* | Yes | Yes | Yes | No | No | No |
| *Felis silvestris* | Yes | Yes | Yes | No | Yes | No |
| *Ficedula albicollis* | No | Yes | No | No | No | Yes |
| *Ficedula hypoleuca* | No | Yes | No | No | No | Yes |
| *Ficedula parva* | No | Yes | No | No | No | No |
| *Ficedula semitorquata* | No | Yes | No | No | No | No |
| *Fringilla montifringilla* | Yes | Yes | No | No | No | No |
| *Galerida cristata* | Yes | No | No | No | No | Yes |
| *Genetta genetta* | Yes | Yes | Yes | Yes | Yes | No |
| *Glaucidium passerinum* | No | Yes | No | No | No | No |
| *Grus grus* | Yes | No | No | Yes | No | No |
| *Gulo gulo* | No | Yes | Yes | Yes | Yes | No |
| *Haliaeetus albicilla* | No | Yes | No | Yes | No | No |
| *Herpestes ichneumon* | Yes | No | No | Yes | Yes | No |
| *Hieraaetus pennatus* | No | Yes | No | No | Yes | No |
| *Hippolais icterina* | No | Yes | No | No | No | No |
| *Hippolais olivetorum* | Yes | Yes | No | No | No | No |
| *Hippolais polyglotta* | No | Yes | No | No | No | No |
| *Jynx torquilla* | Yes | Yes | No | No | No | No |
| *Lagopus lagopus* | No | Yes | No | Yes | No | No |
| *Lanius excubitor* | No | Yes | Yes | No | Yes | No |
| *Lanius minor* | Yes | No | No | No | No | No |
| *Lanius nubicus* | Yes | No | No | No | No | No |
| *Lanius senator* | Yes | No | Yes | No | No | No |
| *Lepus europaeus* | Yes | Yes | Yes | No | No | No |
| *Lepus granatensis* | Yes | Yes | Yes | No | Yes | No |
| *Lepus timidus* | Yes | Yes | Yes | Yes | Yes | No |
| *Loxia curvirostra* | No | Yes | No | No | No | No |
| *Loxia leucoptera* | No | Yes | No | No | No | No |
| *Loxia pytyopsittacus* | No | Yes | No | No | No | No |
| *Loxia scotica* | No | Yes | No | No | No | No |
| *Lullula arborea* | No | Yes | No | No | Yes | No |
| *Luscinia luscinia* | No | Yes | No | Yes | No | No |
| *Lynx lynx* | No | Yes | Yes | Yes | Yes | No |
| *Lynx pardinus* | No | Yes | Yes | Yes | No | No |
| *Martes foina* | Yes | Yes | Yes | Yes | Yes | Yes |
| *Martes martes* | Yes | Yes | Yes | Yes | No | No |
| *Melanocorypha calandra* | Yes | No | No | No | No | No |
| *Meleagris gallopavo* | No | Yes | No | No | No | No |
| *Meles meles* | Yes | Yes | Yes | Yes | No | Yes |
| *Micromys minutus* | Yes | Yes | Yes | Yes | Yes | Yes |
| *Microtus agrestis* | Yes | Yes | Yes | Yes | Yes | Yes |
| *Microtus arvalis* | Yes | No | Yes | No | No | Yes |
| *Microtus duodecimcostatus* | Yes | Yes | Yes | No | No | Yes |
| *Microtus gerbei* | Yes | No | Yes | No | Yes | No |
| *Microtus lusitanicus* | Yes | Yes | Yes | No | Yes | No |
| *Microtus subterraneus* | Yes | Yes | Yes | Yes | Yes | Yes |
| *Milvus migrans* | No | Yes | No | Yes | No | No |
| *Milvus milvus* | No | Yes | No | Yes | No | No |
| *Miniopterus schreibersii* | Yes | Yes | Yes | Yes | Yes | Yes |
| *Muntiacus reevesi* | Yes | Yes | Yes | No | No | No |
| *Mus musculus* | Yes | Yes | Yes | Yes | Yes | Yes |
| *Mus spretus* | Yes | Yes | Yes | No | No | No |
| *Muscardinus avellanarius* | Yes | Yes | No | Yes | No | Yes |
| *Mustela erminea* | Yes | Yes | Yes | Yes | Yes | Yes |
| *Mustela putorius* | Yes | Yes | Yes | Yes | Yes | Yes |
| *Myopus schisticolor* | No | Yes | No | Yes | No | No |
| *Myotis alcathoe* | No | Yes | Yes | Yes | No | Yes |
| *Myotis bechsteinii* | Yes | Yes | Yes | Yes | Yes | Yes |
| *Myotis brandtii* | Yes | Yes | No | Yes | Yes | Yes |
| *Myotis capaccinii* | No | Yes | Yes | Yes | Yes | Yes |
| *Myotis dasycneme* | No | Yes | Yes | Yes | Yes | Yes |
| *Myotis daubentonii* | Yes | Yes | Yes | Yes | Yes | Yes |
| *Myotis emarginatus* | Yes | Yes | Yes | Yes | Yes | Yes |
| *Myotis myotis* | Yes | Yes | Yes | Yes | Yes | Yes |
| *Nucifraga caryocatactes* | No | Yes | No | No | No | No |
| *Numenius arquata* | Yes | No | Yes | Yes | No | No |
| *Numenius phaeopus* | No | Yes | No | Yes | No | No |
| *Numida meleagris* | Yes | No | No | No | No | No |
| *Nyctalus lasiopterus* | Yes | Yes | No | Yes | Yes | Yes |
| *Nyctalus leisleri* | Yes | Yes | Yes | Yes | Yes | Yes |
| *Nyctereutes procyonoides* | Yes | Yes | Yes | Yes | No | Yes |
| *Nycticorax nycticorax* | No | Yes | No | Yes | No | No |
| *Oriolus oriolus* | No | Yes | No | No | No | No |
| *Oryctolagus cuniculus* | Yes | Yes | Yes | No | Yes | Yes |
| *Otis tarda* | Yes | No | Yes | No | No | No |
| *Otus scops* | Yes | Yes | Yes | No | No | No |
| *Pandion haliaetus* | No | Yes | No | Yes | No | No |
| *Passer hispaniolensis* | Yes | No | Yes | No | No | Yes |
| *Pavo cristatus* | No | Yes | No | No | No | No |
| *Perdix perdix* | Yes | No | Yes | No | Yes | No |
| *Perisoreus infaustus* | No | Yes | No | No | No | No |
| *Phylloscopus bonelli* | No | Yes | No | No | No | No |
| *Phylloscopus borealis* | No | Yes | No | No | No | No |
| *Phylloscopus ibericus* | No | Yes | No | No | No | No |
| *Phylloscopus trochiloides* | No | Yes | No | No | No | No |
| *Picoides tridactylus* | No | Yes | No | No | No | No |
| *Picus canus* | No | Yes | No | No | No | No |
| *Pinicola enucleator* | No | Yes | No | No | No | No |
| *Pipistrellus kuhlii* | Yes | Yes | Yes | Yes | Yes | Yes |
| *Pipistrellus nathusii* | Yes | Yes | Yes | Yes | Yes | Yes |
| *Pipistrellus pipistrellus* | Yes | Yes | Yes | Yes | Yes | Yes |
| *Pipistrellus pygmaeus* | Yes | Yes | Yes | Yes | Yes | Yes |
| *Plecotus auritus* | Yes | Yes | No | Yes | Yes | Yes |
| *Pluvialis apricaria* | Yes | No | Yes | Yes | No | No |
| *Podarcis bocagei* | Yes | Yes | Yes | No | Yes | Yes |
| *Pteromys volans* | Yes | Yes | No | No | No | No |
| *Pyrrhula murina* | No | Yes | No | No | No | No |
| *Rana arvalis* | Yes | Yes | Yes | Yes | Yes | No |
| *Rana dalmatina* | Yes | Yes | Yes | Yes | Yes | No |
| *Rana graeca* | No | Yes | Yes | Yes | No | No |
| *Rana temporaria* | Yes | Yes | Yes | Yes | Yes | Yes |
| *Rangifer tarandus* | No | Yes | Yes | Yes | No | No |
| *Rattus rattus* | Yes | Yes | No | Yes | No | Yes |
| *Rhinolophus euryale* | Yes | Yes | Yes | Yes | Yes | Yes |
| *Rhinolophus ferrumequinum* | Yes | Yes | Yes | Yes | Yes | Yes |
| *Rhinolophus hipposideros* | Yes | Yes | Yes | Yes | Yes | Yes |
| *Rhinolophus mehelyi* | Yes | Yes | Yes | Yes | No | No |
| *Salamandra atra* | No | Yes | Yes | Yes | Yes | No |
| *Salamandra salamandra* | No | Yes | Yes | Yes | No | No |
| *Sciurus anomalus* | No | Yes | No | No | No | Yes |
| *Sciurus vulgaris* | Yes | Yes | No | Yes | No | Yes |
| *Scolopax rusticola* | No | Yes | No | No | No | No |
| *Sicista betulina* | Yes | Yes | Yes | Yes | No | No |
| *Sitta krueperi* | No | Yes | No | No | No | No |
| *Sitta whiteheadi* | No | Yes | No | No | No | No |
| *Sorex araneus* | Yes | Yes | Yes | Yes | Yes | Yes |
| *Sorex coronatus* | Yes | Yes | Yes | Yes | No | Yes |
| *Sorex minutus* | Yes | Yes | Yes | Yes | Yes | Yes |
| *Spermophilus citellus* | Yes | No | Yes | No | No | Yes |
| *Streptopelia turtur* | Yes | Yes | No | No | No | No |
| *Strix aluco* | No | Yes | No | No | No | No |
| *Strix nebulosa* | No | Yes | No | No | No | No |
| *Strix uralensis* | No | Yes | No | No | No | No |
| *Sturnus unicolor* | Yes | No | No | No | No | Yes |
| *Suncus etruscus* | Yes | Yes | Yes | Yes | Yes | Yes |
| *Surnia ulula* | No | Yes | No | No | No | No |
| *Sus scrofa* | Yes | Yes | Yes | Yes | Yes | Yes |
| *Sylvia curruca* | Yes | Yes | No | No | No | No |
| *Sylvia hortensis* | Yes | Yes | No | No | No | No |
| *Sylvia melanocephala* | No | Yes | No | No | No | No |
| *Sylvia nisoria* | No | Yes | Yes | No | No | No |
| *Syrmaticus reevesii* | No | Yes | No | No | No | No |
| *Tadarida teniotis* | Yes | Yes | Yes | Yes | Yes | Yes |
| *Talpa europaea* | Yes | Yes | Yes | Yes | No | Yes |
| *Talpa occidentalis* | Yes | Yes | Yes | No | No | Yes |
| *Tamias sibiricus* | No | Yes | No | No | No | Yes |
| *Tarsiger cyanurus* | No | Yes | No | No | No | No |
| *Testudo hermanni* | Yes | Yes | Yes | Yes | No | No |
| *Tetrao urogallus* | No | Yes | No | No | No | No |
| *Tetrax tetrax* | Yes | No | Yes | No | No | No |
| *Tringa ochropus* | No | Yes | No | Yes | No | No |
| *Triturus carnifex* | Yes | Yes | Yes | Yes | No | No |
| *Triturus cristatus* | Yes | Yes | Yes | Yes | Yes | Yes |
| *Triturus dobrogicus* | Yes | Yes | Yes | Yes | No | No |
| *Triturus marmoratus* | Yes | Yes | Yes | Yes | No | No |
| *Turdus iliacus* | No | Yes | No | No | No | No |
| *Turdus pilaris* | No | Yes | No | No | No | No |
| *Turdus torquatus* | No | Yes | No | No | Yes | No |
| *Tyto alba* | Yes | No | Yes | No | No | Yes |
| *Upupa epops* | Yes | No | No | No | No | No |
| *Ursus arctos* | Yes | Yes | Yes | Yes | Yes | Yes |
| *Vespertilio murinus* | Yes | Yes | Yes | Yes | Yes | Yes |
| *Vulpes vulpes* | Yes | Yes | Yes | Yes | Yes | Yes |

1. Histogram of mean coefficient of variation (CV) of model coefficients for each species with mean CV ≥ 0 (*n* = 222). Species with mean coefficient of variation of model coefficients < 0 or larger than the upper quartile of values for the remaining species (1.06) were generally considered to be too unreliable. The plot excludes three outlier species with mean CV > 4.


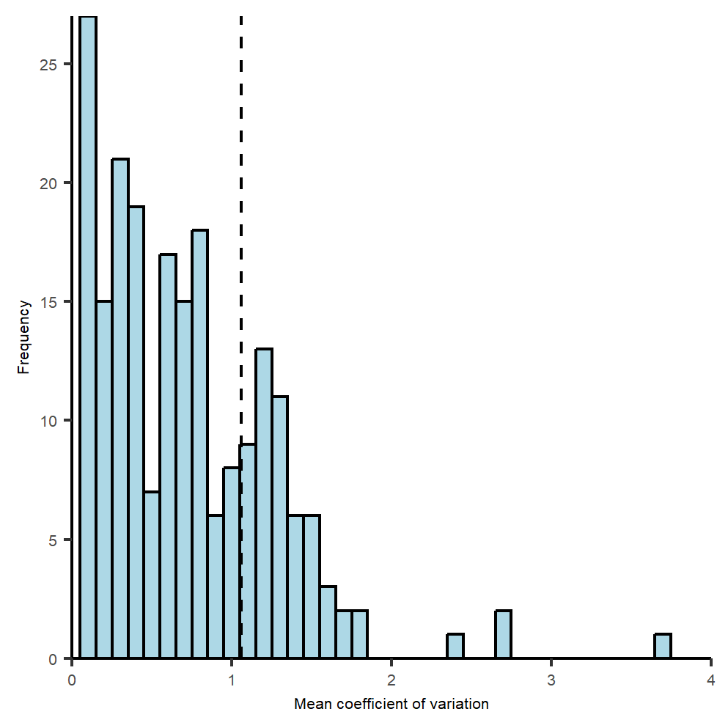


1. Proportion of occurrence points found in each land-use or land-use-intensity class, based on the 1 km^2^ land-system map from Sandström et al. (2023), for cropland and forest species (*n* = 229).


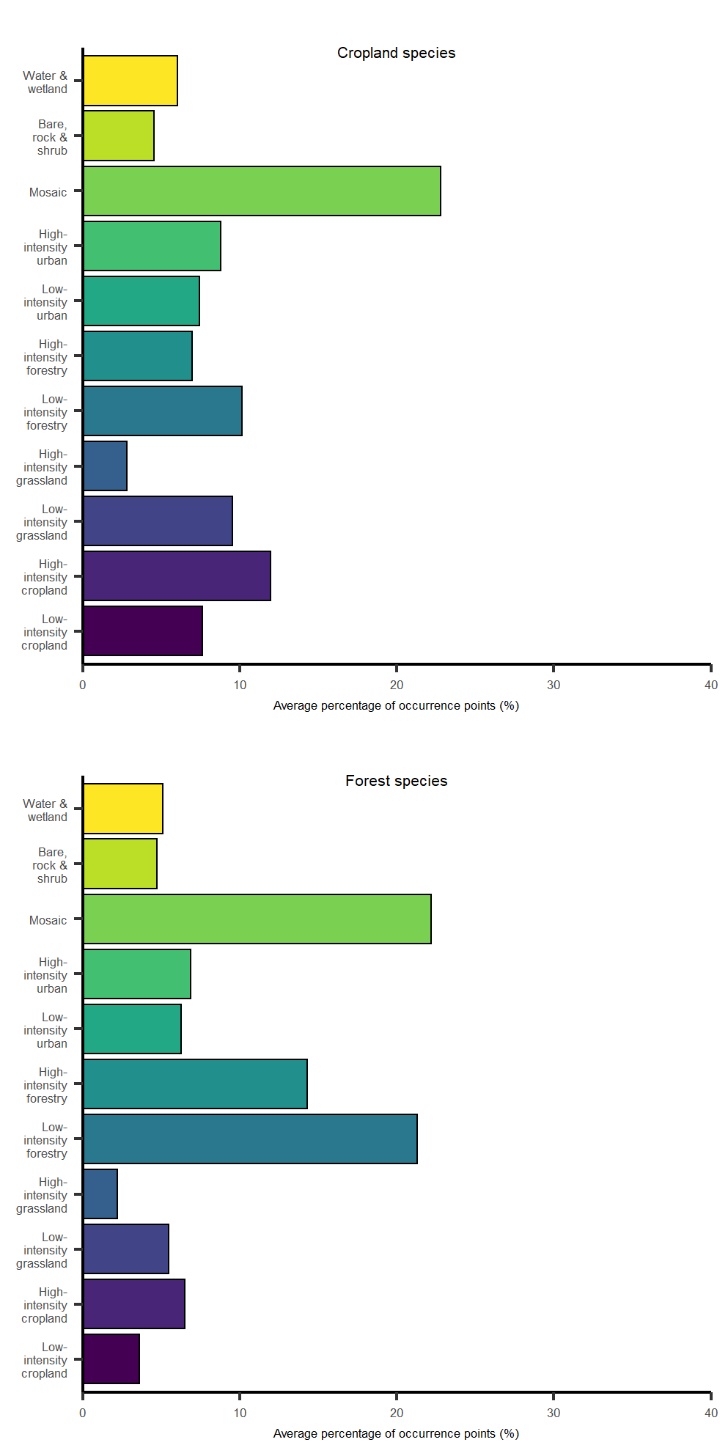


1. AUC results.

Box plot of AUC per species, as the mean AUC of five cross-validation models (*n* = 229).


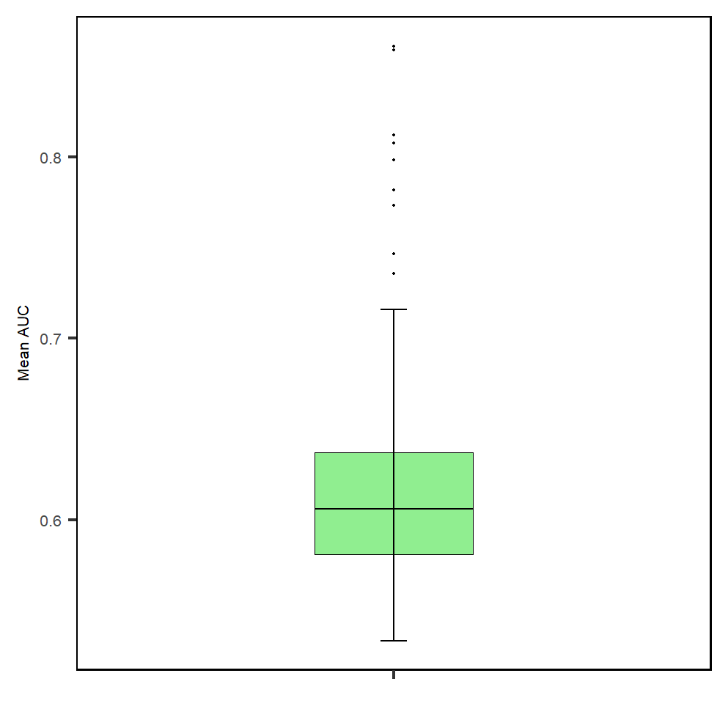


Thirteen species had mean AUC ≥ 0.7, including five threatened species and both critically endangered species (mean AUC for critically endangered species was 0.77). Additionally, eight species had both relatively high model predictive accuracy (mean AUC ≥ 0.7) and relatively stable model coefficient estimates (0 ≤ mean CV ≤ 1.06).

**References**

Dou, Y., Cosentino, F., Malek, Z., Maiorano, L., Thuiller, W. & Verburg, P. H. 2021. A new European land systems representation accounting for landscape characteristics. Landscape Ecology, 36, 2215-2234.

European Union's Copernicus Land Monitoring Service 2018. Imperviousness Density 2018 (raster 10 m), Europe, 3-yearly.

Fourcade, Y., Besnard, A. G. & Secondi, J. 2018. Paintings predict the distribution of species, or the challenge of selecting environmental predictors and evaluation statistics. Global Ecology and Biogeography, 27, 245-256.

IUCN 2024. The IUCN Red List of Threatened Species. Version 2024-1.

Jiménez-Valverde, A. 2012. Insights into the area under the receiver operating characteristic curve (AUC) as a discrimination measure in species distribution modelling. Global Ecology and Biogeography, 21, 498-507.

Lobo, J. M., Jiménez-Valverde, A. & Real, R. 2008. AUC: a misleading measure of the performance of predictive distribution models. Global Ecology and Biogeography, 17, 145-151.

Oostdijk, S., Debonne, N., See, L., Scherpenhuijzen, N. & Verburg, P. 2023. A forest management map for Europe. DRAFT VERSION ed.: DataverseNL.

Roscher, S., Condé, S. & Bailly Maitre, J. 2015. Final database on linkages between species/habitat-types and broad ecosystems. European Topic Centre on Biological Diversity report to the EEA. Paris, France.

Sandström, E., Namasivayam, A., Oostdijk, S., Scherpenhuijzen, N., Debonne, N. & Verburg, P. 2023. Preliminary land system map for Europe. V3 ed.: DataverseNL.

Van Proosdij, A. S. J., Sosef, M. S. M., Wieringa, J. J. & Raes, N. 2016. Minimum required number of specimen

records to develop accurate species distribution models. Ecography, 39, 542-552.
